# Ceftazidime–Avibactam Versus Polymyxin-Based Combination Therapies: A Study on 30-Day Mortality in Carbapenem-Resistant Enterobacterales Bloodstream Infections in an OXA-48-Endemic Region †

**DOI:** 10.3390/antibiotics13100990

**Published:** 2024-10-18

**Authors:** Rıdvan Dumlu, Meyha Şahin, Okan Derin, Özlem Gül, Sedef Başgönül, Rehile Zengin, Çiğdem Arabacı, Funda Şimşek, Serap Gençer, Ayşe Sesin Kocagöz, Ali Mert

**Affiliations:** 1 Department of Infectious Diseases and Clinical Microbiology, Faculty of Medicine, Istanbul Medipol University, 34214 Istanbul, Turkey; meyhasahin@hotmail.com; 2 Epidemiology Doctorate Program, Graduate School of Health Sciences, Istanbul Medipol University, 34815 Istanbul, Turkey; okanderin@gmail.com; 3 Department of Infectious Diseases and Clinical Microbiology, Istanbul Şişli Hamidiye Etfal Training and Research Hospital, 34396 Istanbul, Turkey; ozlemilbay@gmail.com; 4 Department of Infectious Diseases and Clinical Microbiology, Acibadem Maslak Hospital, 34398 Istanbul, Turkey; sedefbasgonul@hotmail.com; 5 Department of Infectious Diseases and Clinical Microbiology, Acibadem Altunizade Hospital, 34662 Istanbul, Turkey; rehile.zengin@acibadem.com; 6 Department of Medical Microbiology, Prof. Dr. Cemil Tascioglu City Hospital, Istanbul Health Science University, 34384 Istanbul, Turkey; dr.c.arabaci@hotmail.com; 7 Department of Infectious Diseases and Clinical Microbiology, Prof. Dr. Cemil Tascioglu City Hospital, Istanbul Health Science University, 34384 Istanbul, Turkey; fundasimsek67@gmail.com; 8 Department of Infectious Diseases and Clinical Microbiology, Faculty of Medicine, Mehmet Ali Aydinlar Acibadem University, 34752 Istanbul, Turkey; serap.gencer@acibadem.com (S.G.); sesin.kocagoz@acibadem.com (A.S.K.); 9 Department of Internal Medicine, Faculty of Medicine, Istanbul Medipol University, 34214 Istanbul, Turkey; alimert@medipol.edu.tr

**Keywords:** ceftazidime–avibactam, polymyxins, OXA-48, carbapenem-resistant enterobacterales, blood stream infections, mortality

## Abstract

**Background**: Ceftazidime–avibactam (CAZ-AVI) is recommended as first-line treatment for Oxacillinase-48 (OXA-48) β-Lactamase-producing carbapenem-resistant Enterobacterales (CRE) infections, while polymyxin-based combination therapies (PBCTs) are used as a last resort when CAZ-AVI is unavailable. Research comparing the effectiveness of CAZ-AVI and PBCT in CRE blood stream infections (CRE-BSIs) is limited, mostly focusing on *Klebsiella pneumoniae* carbapenemase (KPC)-producing isolates. In Turkey, OXA-48 is endemic and OXA-48-Like is common. Therefore, our study aimed to compare the impact of these treatments on 30-day mortality in patients with CRE-BSIs in endemic regions. **Methods**: Retrospective data from January 2019 to May 2023 were collected from four tertiary healthcare centers in Istanbul. Demographic, clinical, and outcome data of ICU patients treated with CAZ-AVI monotherapy or PBCT for CRE-BSIs were analyzed. The effect on 30-day survival was evaluated using Cox regression analysis post propensity score matching (PSM). **Results**: Out of 151 patients, 44.4% (*n*: 67) received CAZ-AVI and 55.6% (*n*: 84) received PBCT. All-cause mortality rates were 20% (*n*: 13) with CAZ-AVI and 36.9% (*n*: 31) with PBCT. Cox regression analysis post PSM indicated CAZ-AVI monotherapy significantly reduced the mortality risk compared to PBCT (HR: 0.16, 95%CI: 0.07–0.37, *p* < 0.001), while age increased the risk (HR: 1.02 per year, 95% CI 1.0–1.04, *p*: 0.01). **Conclusions**: In OXA-48-predominant areas, CAZ-AVI demonstrated significantly lower mortality in patients with CRE-BSIs compared to PBCT. The results were attributed to the pharmacokinetic and pharmacodynamic disadvantages of polymyxins compared to CAZ-AVI, and the impact of age-related physical conditions. Therefore, CAZ-AVI should be the preferred treatment for CRE-BSIs in OXA-48-endemic regions.

## 1. Introduction

Carbapenem-resistant Enterobacterales (CRE) infections remain a global health problem due to their increasing incidence, high mortality rates, and negative impact on health economics [1]. The Centers for Disease Control and Prevention (CDC) reported that the proportion of healthcare-associated CRE infections and related healthcare expenditures have increased over the years [2,3]. Also, in Turkey, carbapenem resistance rates for *Escherichia coli* and *Klebsiella pneumoniae* isolates showed a significant increase from 2017 to 2021 [4].

The Infectious Diseases Society of America (IDSA) 2024 guideline recommends novel beta-lactams for the treatment of CRE. Previously recommended polymyxin-based combination therapies (PBCTs) have been associated with increased mortality and should be avoided [5].

In Turkey, where Oxacillinase-48 (OXA-48) carbapenemase-producing strains are predominant [6], ceftazidime–avibactam (CAZ-AVI) is used as first-line treatment. Nevertheless, in cases where CAZ-AVI is not available due to economic or logistical reasons, or resistance to CAZ-AVI is detected, last-resort treatment options, especially PBCTs, are to be used [7].

CAZ-AVI has been approved by the United States Food and Drug Administration (FDA) and the European Medicines Agency (EMA) for various indications [8,9,10]. Its use in CRE bloodstream infections (CRE-BSIs) is based on limited literature data supporting it. The literature comparing the efficacy of CAZ-AVI and PBCT in CRE-BSIs with real-life data is also limited, and most is on *Klebsiella pneumoniae* carbapenemase (KPC)-producing isolates [11]. In this study, we aimed to compare the effects of the two treatment options on 30-day mortality in patients with CRE-BSIs in Turkey, an endemic area for OXA-48 [12,13], with real-life data. We also investigated the effects of sociodemographic, clinical, and microbiological factors on mortality in patients with CRE-BSIs receiving these treatments.

## 2. Results

Among the 151 patients with CRE-BSIs, 60% (*n*: 91) were male, with a mean age of 62 years (IQR 48–74). Targeted therapy was administered to 44.4% (*n*: 67) of patients with CAZ-AVI and to 55.6% (*n*: 84) of patients with PBCT.

The 30-day mortality rates were 20% (*n*: 13) for CAZ-AVI and 36.9% (*n*: 31) for PBCT. Microbiological eradication rates among patients (*n*: 120) who had follow-up blood cultures after targeted treatment were 100% (*n*: 57) for CAZ-AVI and 53.9% (*n*: 34) for PBCT (*p* < 0.001). Adequate source control was achieved in all patients. No relapse or development of resistance to targeted therapy during the course of treatment was detected in any of the patients.

Demographic and comorbidity characteristics are detailed in Table 1, where older age, higher Charlson Comorbidity Index (CCI) scores, and presence of hematological malignancy were associated with higher mortality rates.

Table 2 presents the clinical and microbiological profiles, treatment regimens, and their outcomes. Mortality was more prevalent among patients with central venous and percutaneous enterogastrostomy (PEG) catheters, septic shock, inotropic agent use, and higher Pittsburgh Bacteremia Index (PBI) scores. The incidence of sepsis alone did not differ significantly between the treatment arms.

*K. pneumoniae* was identified in 98% (*n*: 148) and *E. coli* in 2% (*n*: 3) of the blood cultures. Microbiological eradication was achieved in 60.3% (*n*: 91) of cases. Eradication rates were significantly higher in survivors, though the impact of eradication time on outcomes was not conclusive.

Empirical treatment regimens included piperacillin–tazobactam in 71% (*n*: 60), third- or fourth-generation cephalosporins in 16% (*n*: 14), carbapenems in 80% (*n*: 68), aminoglycosides in 13% (*n*: 11), fluoroquinolones in 12% (*n*: 10), tigecycline in 15% (*n*: 13), and fosfomycin in 6% (*n*: 5) of patients. The mean duration of empirical therapy was 4 days (IQR: 3–5). In survivors, the duration of empirical therapy, as the time between blood culture collection and initiation of targeted therapy, was significantly shorter than in those with a fatal outcome (*p* < 0.001). Additionally, CAZ-AVI as targeted therapy was associated with a significantly lower mortality rate (*p* < 0.001).

Univariate regression analysis identified age as a significant predictor of mortality. Chronic liver disease and a history of hematopoietic stem cell transplantation (HSCT) were also positively correlated with mortality. Higher PBI and CCI scores were associated with increased mortality risk. While sepsis alone did not significantly impact outcomes, septic shock was positively correlated with mortality. Microbiological eradication significantly improved survival outcomes (Table 3).

In multivariate regression analysis following propensity score matching to control for confounding factors, age and the use of CAZ-AVI as targeted therapy emerged as significant predictors of mortality. Each additional year of age increased the risk of mortality 1.03-fold (HR: 1.02, 95% CI 1.0 to 1.04, *p*: 0.01), while the use of CAZ-AVI instead of PBCT statistically reduced mortality risk (HR: 0.16, 95% CI 0.07 to 0.37, *p* < 0.001). No other factors showed a significant impact (Table 3).

**Figure 1:**
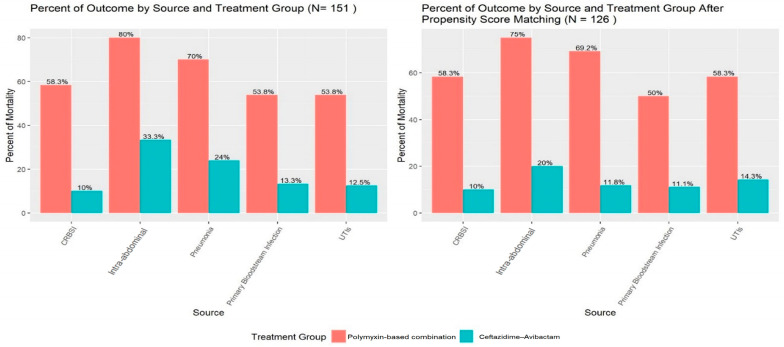
Effect of targeted therapies on outcomes, according to source of infection in total cohort and propensity score-matched groups.

The impact of targeted therapies on outcomes was further analyzed by the source of infection, revealing that CAZ-AVI was associated with lower mortality compared to PBCT across the total cohort and in the propensity score-matched group (Figure 1).

**Figure 2:**
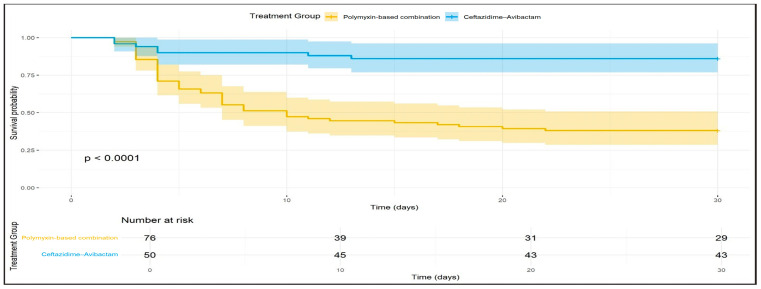
Kaplan–Meier survival analysis for patients receiving Ceftazidime-Avibactam and Polymyxin-based combination therapy for 30 days after treatment initiation.

Kaplan–Meier survival analysis demonstrated that the survival probability at 30 days was significantly higher for patients receiving CAZ-AVI as targeted therapy compared to those receiving PBCT (*p* < 0.0001) (Figure 2).

## 3. Discussion

Our study demonstrates that in Turkey, an OXA-48-endemic region [12,13], CAZ-AVI monotherapy significantly reduces mortality in CRE-BSIs compared to PBCT. It has been reported that there are limited studies in the literature on this issue especially in OXA-48-endemic regions [11]. Hakeam et al. reported lower mortality with CAZ-AVI compared to colistin in 61 patients with CRE-BSIs, most of which were OXA-48 producers, but the difference was not statistically significant [14]. Similarly, Lima et al. found that CAZ-AVI was associated with lower mortality compared to other last-resort therapies in 76 patients with OXA-48-producing CRKP-BSIs, yet the difference lacked statistical significance [15].

Consistent with these two studies, our study observed higher survival rates in patients treated with CAZ-AVI. In contrast, it demonstrated a statistically significant association between CAZ-AVI use and survival. This may be attributed to the multicenter design of our study, a larger cohort, and the use of PSM to control for confounding factors such as age, severity of infection, and comorbidities.

PBCT, which is a last-resort treatment option that was frequently used in the pre-novel antibiotics era in Turkey [16], was associated with an increased mortality risk compared with CAZ-AVI in our study. This is likely due to the pharmacokinetic and pharmacodynamic (PK/PD) differences between CAZ-AVI and polymyxins [17]. Polymyxins have disadvantages, including concentration-dependent activity, a narrow therapeutic window, risk of resistance development during therapy, and insufficient distribution in tissues and body fluids. In contrast, CAZ-AVI offers advantages such as low plasma protein binding, stable volume of distribution, and rapid attainment of bactericidal concentrations [18].

The high prevalence of OXA-48 in Turkey [6] underscores the efficacy of CAZ-AVI in the treatment of CRE-BSIs in this setting. Its broad-spectrum activity against OXA-48-producing strains [5] and its favorable PK/PD properties in treating CRE-BSIs [18] likely contribute to improved survival by providing faster and more effective microbiological clearance. The higher microbiological eradication rate observed with CAZ-AVI and the association of microbiological clearance with survival in univariate analysis further support this argument.

Our study identified older age as another determinant factor for mortality in CRE-BSIs. This finding is consistent with other studies [19,20] in the literature showing age as a risk factor for CRE-BSIs. This may be due to the lower physical and immunological performance of geriatric patients compared to younger populations, coupled with higher rates of hospitalization, ICU admission, and invasive device use.

Univariate regression analysis identified liver disease, history of HSCT, high PBI and CCI scores, and septic shock as mortality-associated factors. These conditions, which exacerbate BSI severity due to compromised immune responses and increased vulnerability to infection, align with those identified in findings from other studies [19,20,21]. This underscores the critical role of utilizing CAZ-AVI as a first-line therapy in high-risk groups. Integrating CAZ-AVI into clinical management strategies for CRE-BSIs may enhance survival outcomes in these susceptible populations.

Compared to the limited literature on the efficacy of CAZ-AVI and PBCT in CRE-BSIs, our study, conducted with a larger cohort, provides statistically significant results in terms of 30-day mortality. This is one of the strengths of the study. Moreover, the use of real-world data increases the clinical applicability of our findings. The retrospective nature of the study and the inability to determine the carbapenemase type are, however, limitations that should be considered when generalizing the results. Nevertheless, these findings highlight the efficacy of CAZ-AVI in high-risk CRE-BSIs in OXA-48-endemic areas and support its prioritization over PBCT in treatment strategies. Future prospective studies that include carbapenemase typing will provide valuable contributions to the literature.

## 4. Material and Methods

### 4.1. Study Design and Setting

This multicenter, observational, retrospective study involved the analysis of intensive care unit (ICU) patients from four tertiary hospitals in Istanbul who developed CRE-BSIs between 1 January 2019 and 30 May 2023. The study protocol, including case definitions, inclusion, and exclusion criteria, was shared with the participating centers. Data on the cases were collected via Microsoft Forms.

Inclusion Criteria:

- Patients aged 18 years or older;
- Admitted to an ICU;
- Blood cultures taken at least 48 h after hospital admission;
- Growth of an Enterobacterales species resistant to at least carbapenem antibiotics in blood cultures;
- Treated with either CAZ-AVI or PBCT for CRE-BSIs.

Exclusion Criteria:

- Polymicrobial growth in blood cultures;
- Mortality within the first 24 h of CRE treatment or less than 24 h of treatment;
- Patients with a concurrent focus of infection other than CRE-BSIs;
- Concurrent use of polymyxins and CAZ-AVI;
- Use of CAZ-AVI in combination therapy;
- Targeted treatment with an agent to which the pathogen was resistant;
- Patients have incomplete or inaccessible data;
- CRE-BSIs that are resistant to only ertapenem among carbapenem-group drugs (since these two regimens are not recommended primarily in their treatment) [5].

Patient data, including sociodemographic characteristics (age and gender), comorbidities, number of days in hospital before diagnosis of bloodstream infection, Charlson Comorbidity Index score [22], Pitt Bacteremia Score [23], presence of sepsis or septic shock, invasive devices used and their types, the focus of infection according to CDC definitions [24], the type of Enterobacterales species, antimicrobial susceptibility patterns, microbiological eradication within the first seven days of targeted treatments, empirical antibiotics used until blood culture results were available, targeted treatments and their duration, and presence of 30-day mortality were collected retrospectively. The study focused on the impact of ceftazidime–avibactam monotherapy and polymyxin-based combination therapy on 30-day mortality.

### 4.2. Ethical Approval

Ethical approval was obtained from the Istanbul Medipol University Clinical Research Ethics Committee (date and decision number: 17 July 2023/E-10840098–772.02–4257). The study was conducted in accordance with the 1964 Helsinki Declaration and Good Clinical Practice guidelines. As this was a retrospective observational study, informed consent from patients or their relatives was waived.

### 4.3. Microbiological Analysis

Blood culture samples were processed using a BacT/ALERT 3D system (bioMérieux, Craponne, France). Positive growth signals were examined via Gram staining and cultured on sheep blood agar, MacConkey agar, and chocolate agar (all from bioMérieux, France). Plates were incubated at 37 °C for 24–48 h. Isolates were identified using a MALDI-TOF MS system (bioMérieux, France). In vitro, antibiotic susceptibility was determined using a VITEK^®^ 2 system (bioMérieux, France) or Phonenix^®^ system (Becton Dickinson, Franklin Lakes, NJ, USA), according to each center’s protocols and based on European Committee on Antimicrobial Susceptibility Testing (EUCAST) criteria [25]. Isolates intermediate or resistant to one or more carbapenem antibiotics were included in the study. Colistin susceptibility was assessed using an MIC-COL kit (Diagnostics, Inc., Galanta, Slovakia). CAZ-AVI susceptibility was determined using the Kirby–Bauer disk diffusion method with ceftazidime–avibactam disks (10/4 microgram) (Oxoid, Basingstoke, UK). Susceptibility thresholds were ≥13 mm for CAZ-AVI and ≤2 µg/mL for colistin.

### 4.4. Treatment Protocol

CAZ-AVI was administered intravenously as a 2 h infusion at a standard dose of 2/0.5 g every 8 h, while colistin and polymyxin B were administered according to the protocol recommendations, which were based dosage on patients’ body mass index [5]. Doses were adjusted for patients with renal impairment during treatments [26,27].

### 4.5. Definitions

CRE-BSIs were diagnosed by the isolation of at least one carbapenem-resistant or increased-exposure-susceptible Enterobacterales strain from blood cultures. However, CRE-BSIs caused by isolates with only ertapenem resistance were excluded from the study since the two treatment options were not the priority treatment [5]. Charlson Comorbidity Index [22] and Pitt Bacteremia Score [23] were calculated on the day of blood culture collection. BSIs were categorized as primary or secondary according to CDC/NHSN surveillance definitions [24].

The presence of sepsis or septic shock on the day of diagnosis was determined using the Sequential Organ Failure Assessment (SOFA) score based on Surviving Sepsis Campaign 2021 criteria [28]. Antibiotic regimens administered during the period from blood culture collection to result confirmation were classified as empirical therapy, while treatments directed against CRE were defined as targeted therapy.

The absence of bacterial growth in blood cultures within the first 7 days of targeted therapy was defined as microbiological eradication. Mortality occurring within 30 days from the start of targeted therapy was considered the outcome. A recurrence of BSI with the same bacterium within 30 days of treatment initiation was defined as a relapse. Clinical improvement was defined as the resolution of all clinical and laboratory findings within 30 days after completing the treatment.

Adequate source control was defined as catheter removal within 48 h of blood culture or drainage via surgical or radiological methods in cases of abscesses or fluid collections.

### 4.6. Statistical Analysis

Data were analyzed using R 4.3.3. [29] and Rstudio [30]. Descriptive statistics were presented as count, percentage, mean, standard deviation, Median, minimum, and maximum values. Statistical hypothesis testing was employed to determine significance based on data type and normality assumptions. Categorical variables were analyzed using Chi-squared or Fisher’s exact tests, while continuous variables were evaluated using either Student’s *t*-test or the Mann–Whitney U test, depending on the distribution. The study was designed to achieve a statistical power of 80% and a significance level of 0.05. 

To mitigate potential selection bias arising from baseline differences between the treatment groups (“PBCT” and “CAZ-AVI”), we employed propensity score matching (PSM) with the “MatchIt” package [31]. The propensity score, which estimates the probability of receiving a specific treatment based on observed covariates, was calculated using logistic regression with a probit link function. The matching process utilized a nearest-neighbor algorithm with a caliper, aiming to pair individuals from the two groups with similar propensity scores. The effectiveness of the matching was assessed by examining the standardized mean differences in covariates before and after matching. Details of the propensity score matching study are provided in the Supplementary Material (Supplement Text S1).

Following successful propensity score matching, we conducted survival analyses to investigate the association between the treatment type and the outcome of interest (30-day mortality). Both univariable and multivariable Cox proportional hazards regression models were employed. The univariable analyses assessed the individual effects of each predictor variable on the outcome, while the multivariable model incorporated multiple predictors simultaneously to evaluate their independent contributions, adjusting for potential confounding effects. The proportional hazards assumption was verified to ensure the validity of the Cox models. The hazard ratios (HRs) and their corresponding 95% confidence intervals were reported to quantify the risk associated with each predictor. 

## 5. Conclusions

In conclusion, our study shows that in an OXA-48-endemic region, CAZ-AVI monotherapy significantly reduces 30-day mortality in CRE-BSIs compared to PBCT. We believe that prioritizing CAZ-AVI over last-resort therapies in this specific context and improving its availability is crucial, especially in regions where access to novel antibiotics is limited.

## Figures and Tables

**Table 1:** The effect of demographic data and case backgrounds on the outcome.

Variable	Overall, *n* = 151 (%) ^1^	Survived, *n* = 85 (55.5%) ^1^	Death, *n* = 66 (44.5%) ^1^	*p*-Value ^2^
Patient-Related Factors				
Age (Median (IQR))	62(48–74)	55 (44–67)	68 (56–76)	<0.001
Sex (Male)	91 (60.3)	53 (62)	38 (58)	0.6
Days between BC and hospital admission	20 (10–34)	21 (12–33)	17 (10–35)	0.4
Days between BC and ICU admission	9 (2–25)	10 (3–27)	9 (2–24)	0.6
CRE colonization	53 (35.1)	31 (36)	22 (33)	0.7
History of CRE infection	115 (76.2)	69 (81)	46 (70)	0.1
COVID-19 co-infection	37 (24. 5)	19 (22)	18 (27)	0.5
Comorbidities				
CCİ (Median (IQR))	4 (2–6)	3 (2–6)	5 (3–7)	0.006
Cardiovascular Diseases	72 (47.7)	38 (45)	34 (52)	0.4
Diabetes Mellitus	53 (35.1)	25 (29)	28 (42)	0.1
Pulmonary Diseases	32 (21.2)	19 (22)	13 (20)	0.7
Neurological Diseases	42 (27.8)	23 (27)	19 (29)	0.8
Hematologic Diseases	18 (11.9)	5 (6)	13 (20)	0.009
Malignancy	37 (24.5)	22 (26)	15 (23)	0.7
Chronic Renal Failure	24 (15.9)	15 (18)	9 (14)	0.5
Auto-Immune Diseases	9 (6.0)	5 (6)	4 (6)	>0.9
Liver Diseases	6 (4.0)	1 (1)	5 (8)	0.086
SOT	6 (4.0)	3 (4)	3 (5)	>0.9
HSCT	8 (5.3)	2 (2)	6 (9)	0.080

^1^: *n* (%) and Median (IQR 25–75) results. ^2^: Pearson’s Chi-squared test; Wilcoxon rank sum test; Fisher’s exact test. BC: day of blood culture collection; ICU: intensive care unit; CRE: carbapenem-resistant Enterobacterales; CCI; Charlson Comorbidity Index; SOT: Solid Organ Transplantation; HSCT: hematopoietic stem cell transplantation.

**Table 2:** The effect of clinical and microbiological features and treatments on the outcome.

		Characteristics by Outcome			
Variable	*n*	Overall, *n* = 151 ^1^	Survived, *n* = 85 (56.3%) ^1^	Death, *n* = 66 (43.7%) ^1^	*p*-Value ^2^
İnvasive procedures in ICU	151 (100.0)				
Mechanical ventilation		138 (91.4)	75 (88)	63 (95)	0.12
Central venous catheter		137 (90.7)	73 (86)	64 (97)	0.020
Urinary catheter		148 (98)	82 (96)	66 (100)	0.3
PEG		21 (13.9)	7 (8)	14 (21)	0.022
İnotropic infusion		126 (83.4)	64 (75)	62 (94)	0.002
RRT		47 (31.1)	23 (27)	24 (36)	0.2
COVID-19 co-infection		37 (24. 5)	19 (22)	18 (27)	0.5
Source of infection	151 (100.0)				0.4
Primary BSI		28 (18.5)	19 (22%)	9 (17%)	
Secondary BSI		123 (81.5)	66 (78%)	57 (86%)	
Pneumonia		55 (36.4)	28	27	
Catheter-related BSI		22 (14.6)	14	8	
Urinary tract infection		21 (13.9)	13	8	
Intra-abdominal infection		16 (10.6)	6	10	
Skin and soft tissue infection		6 (4)	4	2	
Central nervous system infection		2 (1.3)	1	1	
Osteomyelitis		1 (0.7)	0	1	
Severity of infection	151 (100.0)				
Pitt score (Median (IQR))		8 (6–9)	6 (5–8)	8 (7–12)	<0.001
Sepsis		142 (94)	77 (91)	65 (98)	0.078
Septic shock		122 (80)	60 (71)	62 (94)	<0.001
Microbiological features	151 (100.0)				
Type of CRE	151 (100.0)				0.6
*K. pneumoniae*		148 (98)	84 (98.8)	64 (97)	
*E. coli*		3 (2)	1 (1.2)	2 (3)	
Sterilization of control BC	151 (100.0)	91 (60.3)	75 (88.2)	16 (24.2)	<0.001
Sterilization achieved time (days) (Median (IQR))	91 (60.3)	5 (3–5)	5 (3–5)	5 (4.5–7)	0.15
Empiric treatment duration (days) (Median (IQR))	151 (100)	4 (3–5)	3 (3–4)	5 (4–6)	<0.001
Anti-CRE treatment	151 (100)				
Ceftazidime–avibactam		67 (44.4)	54 (63.5)	13 (19.7)	<0.001
Polymyxin-based combination therapy		84 (55.6)	53 (62.4)	31 (47)	0.8
Colistin		61 (40.8)	23 (27.1)	38 (57.6)	
Polymyxin B		23 (15.2)	8 (9.4)	15 (22.7)	
Agent combined polymyxins		84 (55.6)	53 (62.4)	31 (47)	0.8
High-dose meropenem		47 (60)	17 (32)	30 (96.8)	0.7
Aminoglycosides		13 (15.4)	5 (9.4)	8 (25.8)	>0.9
Fluoroquinolones		4 (4.8)	2 (3.8)	2 (6.5)	0.5
Tigecycline		40 (47.6)	15 (28.3)	25 (80.6)	>0.9
Fosfomycin (IV)		10 (11.9)	1 (1.9)	9 (29)	0.083

^1^: *n* (%) and Median (IQR 25–75) results. ^2^: Pearson’s Chi-squared test; Wilcoxon rank sum test; Fisher’s exact test. ICU: intensive care unit; CRE: carbapenem-resistant Enterobacterales; PEG: Percutaneous Endoscopic Gastrostomy Tube; RRT: Renal Replacement Therapy; BC: Day Blood Culture Received; IV: Intravenous.

**Table 3:** Investigation of the effect of demographic data and history of the cases on the outcome.

Parameters		Univariate Cox Analysis			Multivariate Cox Analysis		
		HR	%95 CI	*p*-Value	HR	%95 CI	*p*-Value
Age		1.02	1.00–1.04	0.010	1.03	1.01–1.04	0.006
Sex (Male)		0.91	0.53–1.56	0.7	1.08	0.60–1.95	0.8
Pitt Bacteremia Score		1.15	1.05–1.25	0.002	-	-	-
CCI		1.12	1.03–1.21	0.005	-	-	-
Presence of mechanical ventilators		1.95	0.61–6.25	0.3	1.06	0.29–3.83	>0.9
Central venous catheter		2.80	0.68–11.5	0.2	2.33	0.51–10.7	0.3
PEG		1.51	0.76–3.01	0.2	0.93	0.43–1.99	0.8
RRT		1.35	0.76–2.40	0.3	1.45	0.76–2.76	0.3
Sepsis		4.80	0.66–34.7	0.12	-	-	-
Septic shock		3.71	1.34–10.3	0.012	-	-	-
CRE colonization		1.04	0.59–1.84	0.9	1.02	0.53–1.96	>0.9
Microbiological eradication		0.08	0.04–0.15	<0.001	-	-	-
History of Chronic Disease	Diabetes Mellitus	1.29	0.75–2.22	0.4	1.29	0.59–2.83	0.5
	Cardiovascular Disease	1.16	0.68–1.98	0.6	0.79	0.39–1.61	0.5
	Hematological Disease	2.02	0.91–4.48	0.082	2.58	0.90–7.37	0.078
	Malignancy	1.04	0.56–1.95	0.9	1.53	0.66–3.55	0.3
	Liver Disease	5.03	1.79–14.1	0.002	-	-	-
	HSCT	2.94	1.06–8.16	0.039	-	-	-
Ceftazidime–avibactam monotherapy		0.16	0.07–0.37	<0.001	0.12	0.05–0.33	<0.001

HR: hazard ratio; CI: confidence interval; CCI: Charlson Comorbidity Index; PEG: Percutaneous Endoscopic Gastrostomy Tube; RRT: Renal Replacement Therapy; CRE: carbapenem-resistant Enterobacterales; HSCT: hematopoietic stem cell transplantation.

## Data Availability

The datasets generated during and/or analyzed during the current study are not publicly available due to administrative reasons but are available from the corresponding author on reasonable request.

## Supplementary Materials

The following supporting information can be downloaded at https://www.mdpi.com/article/10.3390/antibiotics13100990/s1. Figure S1: Balance of Covariates Before and After Propensity Score Matching. Figure S2: Distribution of Propensity Scores Before and After Matching. Figure S3: Histograms of Propensity Scores for Treated and Control Groups Before and After Matching. Supplement Text S1: Detailed statistical information about probability score matching analysis.
