# Supplementary material for: Ceftazidime–Avibactam Versus Polymyxin-Based Combination Therapies: A Study on 30-Day Mortality in Carbapenem-Resistant Enterobacterales Bloodstream Infections in an OXA-48-Endemic Region
Source: Antibiotics (Basel). 2024 Oct 18;13(10):990. doi: 10.3390/antibiotics13100990 (PMC11505437; doi:10.3390/antibiotics13100990)
Supplement: Supplementary file 1 [file antibiotics-13-00990-s001.zip › antibiotics-3221917-supplementary.pdf]

Supplement Text S1

Propensity Score Matching Study

Our propensity score matching analysis was conducted using the MatchIt package in R. We aimed to evaluate the effect of treatment on outcomes after adjusting for the covariates: age, sex, mechanical ventilation status, septic shock status, COVID-19 status, Charlson comorbidity index, and meropenem antimicrobial susceptibility test result.

We employed nearest neighbour matching with an average treatment effect on the treated (ATT) estimation approach to match treated individuals to control individuals with similar propensity scores, thereby reducing selection bias due to observed confounders. The propensity scores were estimated using a logistic regression model with a probit link function, which is suitable for binary outcomes.

Prior to matching, there was a considerable imbalance in the distribution of propensity scores between treated and control groups, indicated by a high standardized mean difference of 2.3579. Other covariates such as "*meropenem_resistant*" also exhibited notable imbalances (standardized mean difference = 0.1667).

Post-matching, the standardized mean differences were significantly reduced, demonstrating improved balance across all covariates. For instance, the standardized mean difference for the distribution of propensity scores was reduced to 0.1074, indicating that the matching process successfully created a more comparable control group. The binary covariates, such as "*sex_female*", "*mech_vent_no*", and "*mem_resistant*", achieved perfect balance with a standardized mean difference of 0.0000, suggesting no residual bias in the distribution of these covariates between the matched treatment and control groups.

The final matched dataset comprised 67 treated and 67 control observations, with 17 control observations being unmatched and thus excluded from the matched analysis. This matching process enabled a more accurate estimation of the treatment effect by ensuring comparability between treatment groups.

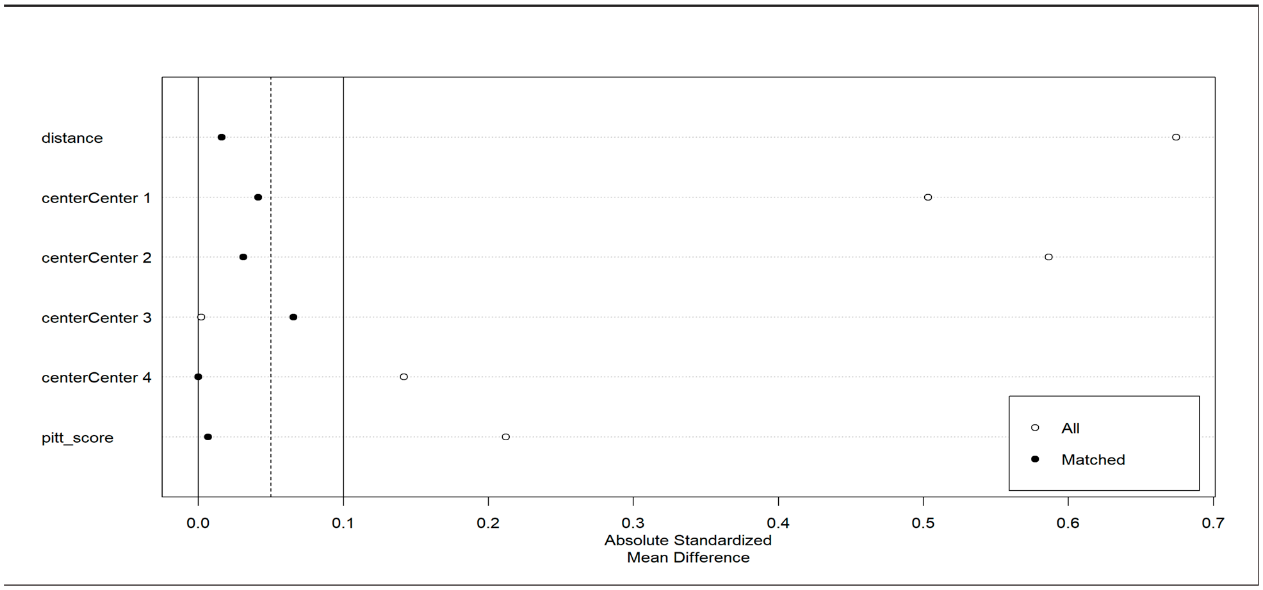

Figure S1. Balance of Covariates Before and After Propensity Score Matching.

This plot illustrates the absolute standardized mean differences (ASMD) for key covariates before and after propensity score matching. The open circles represent the ASMDs for the original unmatched data, while the filled circles represent the ASMDs for the matched data. Covariates include distance, center (with categories Center 1, Center 2, Center 3, and Center 4), and pitt_score. A dashed vertical line at ASMD of 0.1 indicates the threshold below which covariate balance is considered acceptable. The matching process significantly improved the balance of covariates between the treatment groups.

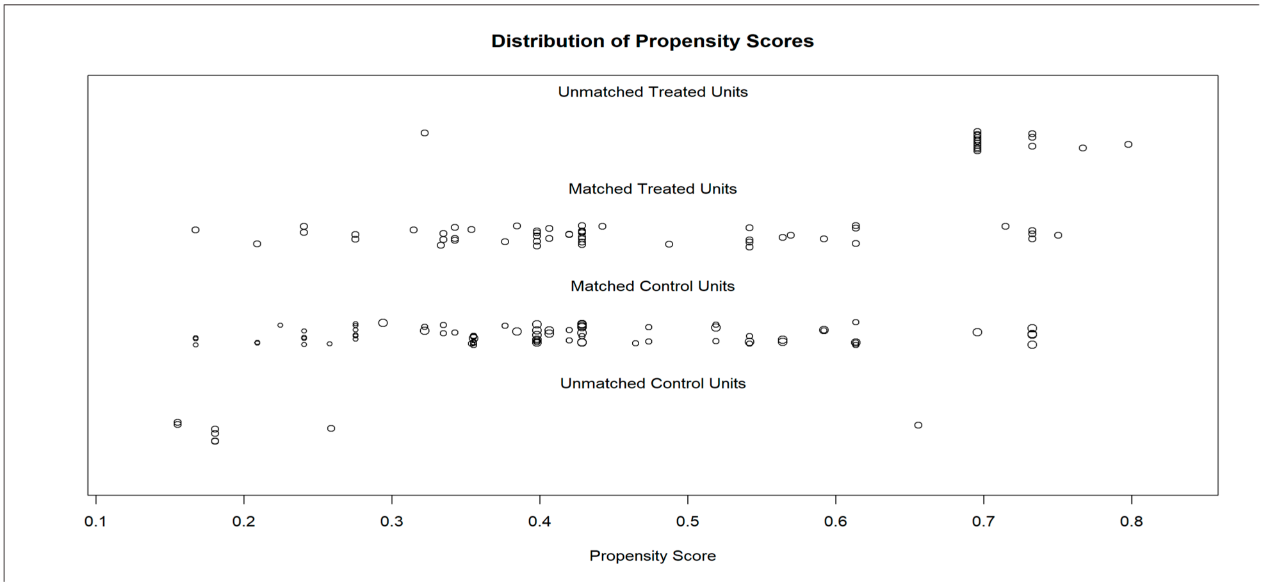

Figure S2. Distribution of Propensity Scores Before and After Matching

This figure shows the distribution of propensity scores for treated and control units before and after matching. The scatter plot displays four groups: unmatched treated units, matched treated units, unmatched control units, and matched control units.

Propensity scores range from 0.1 to 0.8, illustrating the overlap and balance between treated and control groups achieved through the matching process.

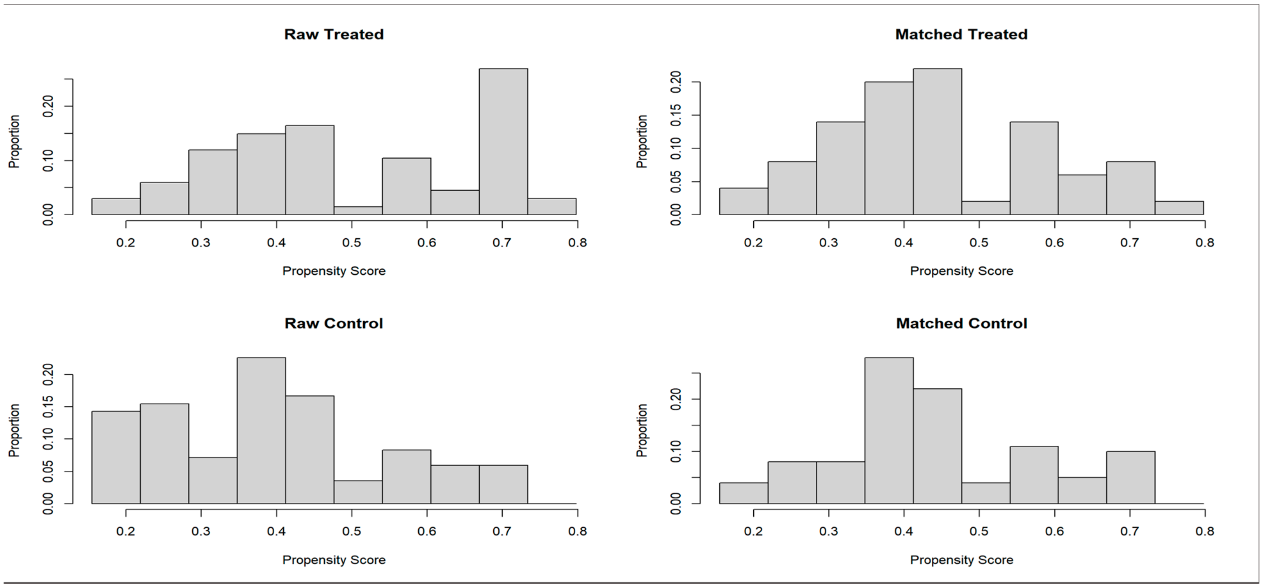

Figure S3. Histograms of Propensity Scores for Treated and Control Groups Before and After Matching.

The x-axis represents the propensity score values, and the y-axis represents the proportion of subjects within each bin of the propensity score. The left panels show the raw (unmatched) data, and the right panels show the matched data. The top panels represent the treated group, while the bottom panels represent the control group.
